# Dynamic measurement of pennation angle of gastrocnemius muscles during contractions based on ultrasound imaging

**DOI:** 10.1186/1475-925X-11-63

**Published:** 2012-09-03

**Authors:** Yongjin Zhou, Ji-Zhou Li, Guangquan Zhou, Yong-Ping Zheng

**Affiliations:** 1 Shenzhen Institutes of Advanced Technology, Chinese Academy of Sciences, Shenzhen, China; 2 The Shenzhen Key Laboratory for Low-cost Healthcare, Shenzhen, China; 3 Interdisciplinary Division of Biomedical Engineering, The Hong Kong Polytechnic University, Hong Kong, China; 4 College of Mathematics and Econometrics, Hunan University, Changsha, China

**Keywords:** Ultrasound image, Pennation angle, Hough transform, Sonomyography, SMG, Electromyography, EMG, Gastrocnemius muscle, Orientation

## Abstract

**Background:**

Muscle fascicle pennation angle (PA) is an important parameter related to musculoskeletal functions, and ultrasound imaging has been widely used for measuring PA, but manually and frame by frame in most cases. We have earlier reported an automatic method to estimate aponeurosis orientation based on Gabor transform and Revoting Hough Transform (RVHT).

**Methods:**

In this paper, we proposed a method to estimate the overall orientation of muscle fascicles in a region of interest, in order to complete computing the orientation of the other side of the pennation angle, but the side found by RVHT. The measurements for orientations of both fascicles and aponeurosis were conducted in each frame of ultrasound images, and then the dynamic change of pennation angle during muscle contraction was obtained automatically. The method for fascicle orientation estimation was evaluated using synthetic images with different noise levels and later on 500 ultrasound images of human gastrocnemius muscles during isometric plantarflexion.

**Results:**

The muscle fascicle orientations were also estimated manually by two operators. From the results it’s found that the proposed automatic method demonstrated a comparable performance to the manual method.

**Conclusions:**

With the proposed methods, ultrasound measurement for muscle pennation angles can be more widely used for functional assessment of muscles.

## Background

Muscle fascicle pennation angle (PA), muscle thickness (MT) and fiber length (FL) and their dynamic changes during muscle contraction have become important measures for skeletal muscle studies using ultrasound, for example [1-5]. The change of PA and FL over the time can form signals, representing architectural muscle behavior under contraction, similar to the change of MT, which has been defined as sonomyography (SMG) [6]. SMG can provide muscle functional information complementary to electromyography (EMG) and torque signals [7,8]. In previous studies, pennation angles were conventionally detected manually in ultrasound images of muscles, for example [2,9-11], and this greatly affects the wider applications of this parameter, particularly for the study of dynamic muscle contraction [12-14].

Recently, a number of studies have been reported for the automatic estimation of muscle fascicle orientation and pennation angle using revoting Hough transform (RVHT) [15,16], Radon transform [17,18] or features-separability filtering [19].

**Figure 1:**
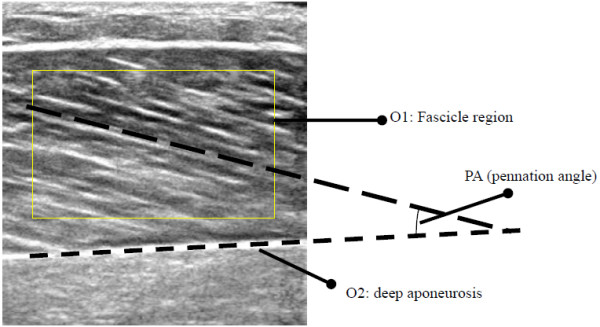
Typical sonogram of the medial gastrocnemius muscle.

However, these proposed methods could only provide the orientation of aponeuroses or some individual fascicles in ultrasound images with quality contrast. During muscle contraction, the fascicles may not only change in orientation and length, but also their contrasts in ultrasound images, and this makes the orientation estimation of individual fascicles not so reliable using the above methods. For a typical ultrasound image of muscle, such as the gastrocnemius muscle as shown in Figure 1, the process of estimating the pennation angle involves the measurement of orientations of the aponeuroses and the fascicle region between aponeuroses. The aim of this study is to use RVHT to estimate the aponeurosis orientation and to use the dominant texture orientation of the fascicle region to represent the fascicle orientation. Note that in estimation of the fascicle orientation, the information from a region rather than several individual fascicles are employed. Then the pennation angle is estimated as the difference between orientation of the fascicle and the deep aponeurosis. Since the measurement is automatic, it can be used to measure the pennation angle in each ultrasound image collected during muscle contraction.

## Methods

### Estimation of deep aponeurosis orientation

The estimation of the deep aponeurosis orientation (O2 in Figure 1) was based on the methods that we have reported earlier, which included using Gabor filtering to enhance ultrasound images and RVHT to estimate the orientation [15,16]. This process was repeated for each frame of ultrasound images, and the orientation change was recorded using RVHT method [15,16].

### Estimation of dominant fascicle orientation of selected region

To compute the dominant orientation of the selected region of fascicle in ultrasound images, such as the O1 region shown in Figure 1, the local orientation field was acquired in a multi-step process for each pixel. The original region,$I\left(i,j\right)$, was first smoothed with a Gaussian filter. Then at each pixel, the gradients, $\partial_{x}\left(i,j\right)$and$\partial_{y}\left(i,j\right)$, were computed and then the primary local orientation for each pixel was computed using Rao’s scheme [20]:

(1) $$Sum_{x}\left(i,j\right)=\sum_{u=i-w/2}^{i+w/2}\sum_{v=j-w/2}^{j+w/2}2\partial_{x}\left(u,v\right)\partial_{y}\left(u,v\right)$$

(2) $$Sum_{y}\left(i,j\right)=\sum_{u=i-w/2}^{i+w/2}\sum_{v=j-w/2}^{j+w/2}\left(\partial_{x}^{2}\left(u,v\right)-\partial_{y}^{2}\left(u,v\right)\right)$$

(3) $$\theta \left(i,j\right)=\frac{\pi }{2}+\frac{1}{2}\tan^{-1}\left(\frac{Sum_{y}\left(i,j\right)}{Sum_{x}\left(i,j\right)}\right)$$

where $\theta \left(i,j\right)$ is the least square estimation of the orientation at pixel$\left(i,j\right)$ and *w* x *w* defined its neighborhood area involved.

The reliability coefficient of the orientation field [20] was measured by

(4) $$R\left(i,j\right)={\left(\partial_{\text{i}}^{2}+\partial_{\text{j}}^{2}\right)}^{1/2}\frac{\sum_{\left(k,l\right)\in \Gamma }\left|{\left(\partial_{k}^{2}+\partial_{l}^{2}\right)}^{1/2}*\cos\left(\theta \left(i,j\right)-\theta \left(k,l\right)\right)\right|}{\sum_{\left(k,l\right)\in \Gamma }{\left(\partial_{k}^{2},+,\partial_{l}^{2}\right)}^{1/2}}$$

where *Γ* is a small neighboring region of the pixel$\left(i,j\right)$, and its size is related to the local frequency of strongly oriented patterns. The reliability coefficient here is a number between 0 and 1, and its two extremities, 0 and 1, correspond to the isotropic region and the strongly oriented pattern, respectively. To estimate the dominant orientation of the selected region, we used the median value of the orientations for each pixel in the region, as long as its reliability coefficient was larger than an empirically pre-defined threshold, 0.6 in this paper. The fascicle pennation angle was computed as the difference between the dominant orientation of the selected fascicle region O1 and the deep aponeurosis orientation O2.

### Estimation of the dynamic changes of pennation angle

**Figure 2:**
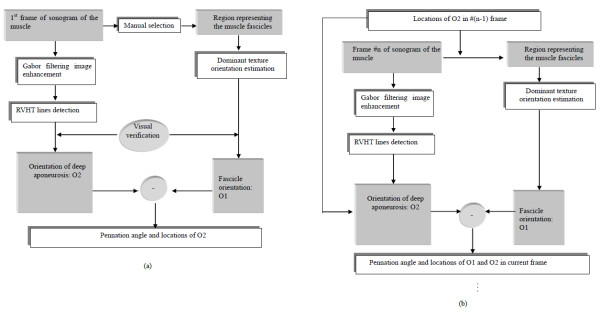
**Procedures of estimating the pennation angles.** (**a**) Procedure of estimating the pennation angle in the first frame of the ultrasound image sequence and (**b**) Procedure of estimating the pennation angle in the subsequent frames following the first frame.

In the first image of a series of ultrasound images collected during muscle contraction, the interested fascicle region was manually selected and its orientation was calculated based on the proposed method. Meanwhile, the orientation and location of the interested deep aponeurosis were detected, with visual verification. The procedure for processing the 1^st^ frame and calculating the pennation angle is described in Figure 2a. Since the location and orientation of the deep aponeurosis would change little during the contraction, its change between two frames was confined within a certain range. This helps to track consistently the same aponeurosis during the muscle contraction. For the selected fascicle region, the dominant orientations at the same region of interest in the subsequent frames were computed using the same procedures as those for the first frame, as shown in Figure 2b. Thus, the dynamic changes of the pennation angle could be automatically estimated frame by frame as the muscle contracts and relaxes.

## Results

### Evaluation of the texture dominant orientation method using synthetic images

**Figure 3:**
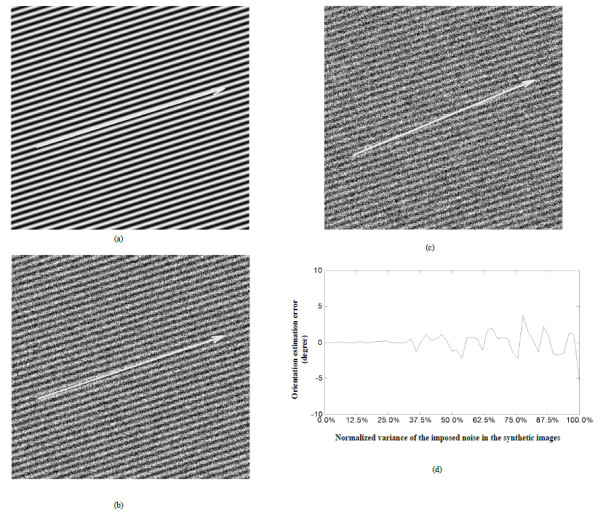
**Analysis of results on synthetic images.** Analysis of results on synthetic images with different noise levels, the actual orientation is arctangent of 1/3 (18.4 degrees), the arrows overlaid on (**a**-**c**) stand for the orientations estimated. (**a**) Synthetic image, noise: mean = 0, normalized variance = 0%; (b) Synthetic image, noise: mean = 0, normalized variance = 50%; (**c**) Synthetic image, noise: mean = 0, normalized variance = 100%. (**d**) Errors of the estimated orientation compared to the actual value.

All codes used in this section were written in Matlab. The method was first evaluated using a set of synthetic images with different levels of noises. The image was constructed to simulate a region of muscle fascicle by first constructing an image with only horizontally parallel patterns and each column of which was a sinusoid waveform in gray level with peak and valley of 250 and 125 (in a range between 0 to 255) respectively, then patterns were rotated by 18.4 degrees (arctangent of 1/3). After rotation, different levels of noises were imposed onto them using Matlab function “imnoise”. The noise had a mean of 0 and normalized variance from 0% to 100%. To reduce errors caused by the digitization operations in synthetic image generation, the synthetic images were rotated by −18.4 degrees (− arctangent of 1/3) and the orientation were then estimated using the proposed method and compared to the altered ground truth of 0°. Figure 3a to 3c show typical synthetic images with different noise levels, with the normalized variance of 0%, 50% and 100%. The error of estimated results in comparison with the actual value was shown in Figure 3d. The results indicated that the proposed orientation method was robust for a noise level smaller than 30%. As the increase of the noise level from 50% to 100%, the estimation error was relatively stable, though there was a small variance of within +/− 5 degrees in comparison with the actual value.

### Evaluation of the automatic estimation method for continuous pennation angle changes

**Figure 4:**
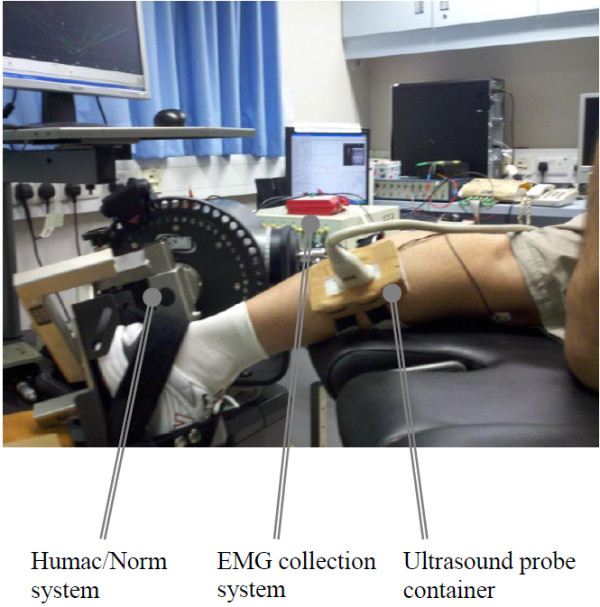
**The experimental setup.** The experimental setup including the torque, EMG and ultrasound image data collection modules.

A dynamometer (Humac/Norm Testing and Rehabilitation System, Computer Sports Medicine, Inc., Massachusetts, USA) was used to assist the subject to conduct the designed contraction pattern of isometric plantarflexion. A real-time B-mode ultrasonic scanner (EUB-8500, Hitachi Medical Corporation, Tokyo, Japan) with an electronic linear array probe (L53L, Hitachi Medical Corporation, Tokyo, Japan) was used to obtain ultrasound images of muscles. The ultrasound probe was aligned perpendicularly to the gastrocnemius muscle belly using a custom-designed foam container with fixing straps and the long axis of the ultrasound probe was arranged parallel to the long axis of the gastrocnemius muscle, and then a very generous amount of ultrasound gel was applied to secure acoustic coupling between the probe and skin during the muscle contractions, as shown in Figure 4. The probe was adjusted to optimize the contrast of muscle fascicles in ultrasound images. The B-mode ultrasound images were displayed in real time and digitized by a video card (NI PCI-1411, National Instruments, Austin, USA) at a rate of 25 frame/s for later analysis.

Surface EMG signals were collected from the gastrocnemius muscle using bipolar Ag-AgCl electrodes (Axon System, Inc., NY, USA), amplified by a multiple channel amplifier (RM6280 Multi-Channel Biosignal Collection and Processing System, Chengdu Instrument Company, Chengdu, China), with a gain of 2000, filtered separately by 10–400 Hz, 5-100 Hz band-pass analog filters within the amplifier, and then digitized by a 12-bit data acquisition card (NI-DAQ 6024E, National Instruments Corporation, Austin, TX, USA) with a sampling rate of 1 kHz. Ultrasound image sequences, surface EMG and torque signals were simultaneously collected and stored by a custom-made program for ultrasonic measurement of motion and elasticity (UMME, http://www.tups.org).

One young male subject (age 29, body weight 67Kg and height 172 cm) participated in the test to demonstrate the feasibility of the method. Human subject ethical approval was obtained from the relevant committee in the authors’ institution, and informed consent was obtained from the subject prior to the experiment. The testing position of the subject was in accordance with the Humac/Norm User’s Guide. The subject was instructed to put forth his maximal effort of isometric plantarflexion for a period of 3 s with verbal encouragement provided. The maximal voluntary contraction (MVC) was defined as the highest value of torque recorded during the entire isometric contraction. A rest of 5 min was allowed before the subject performing another MVC test. The MVC torque was then calculated by averaging the two recorded highest torque values from the two tests. The subject was instructed to generate a torque waveform in rough sinusoid shape, up to 90% of his MVC, using ankle plantarflexion movements in prone position.

**Figure 5:**
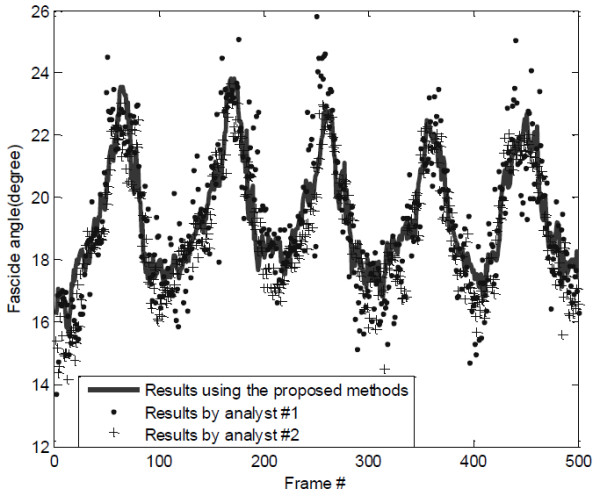
**Comparison of Fascicle orientations estimated using manual and automatic methods.** Fascicle orientations estimated manually by the two operators and the results using the proposed automatic method. The x-axis is equivalent to 0–20 s in imaging time.

**Figure 6:**
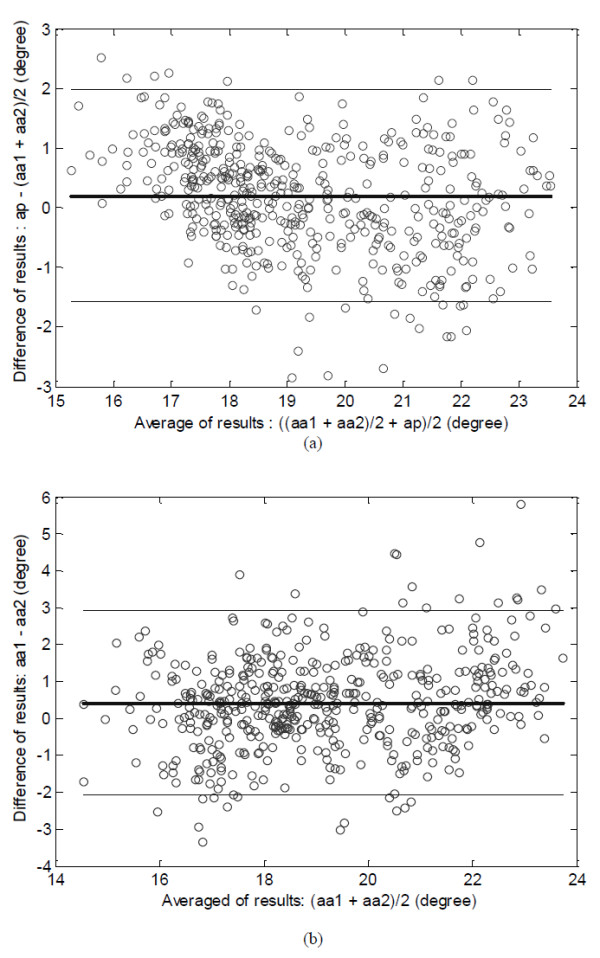
**The Bland-Altman plot.** (**a**) The Bland-Altman plot of the mean fascicle orientations estimated manually by the two operators, (*aa1* + *aa2)/2*, and the results obtained using the proposed automatic method, *ap*. (**b**) The Bland-Altman plot of the results obtained by the two operators. The bold horizontal line represents the mean and the thin lines indicates the mean + 1.96*SD and mean-1.96*SD levels. SD represents standard deviation.

The first frame of the collected ultrasound image sequence (500 frames) for the lateral gastrocnemius in plantarflexion motion was shown in Figure 1, and the estimated changes of orientation of fascicle region O1 using the proposed methods, denoted by *ap*, was shown in Figure 5. To evaluate the automatic measurement results, the fascicle orientations in all the frames of ultrasound images were manually estimated by two operators, who had the experiences in ultrasound imaging of muscles. Their estimations were blinded from each other, and their results were denoted by *aa1* and *aa2* and shown in Figure 5*.* It was observed that there were good correlations among the results obtained by the two operators and estimated by the proposed automatic method. The Bland-Altman plots [21] of the results showed that there was a good agreement between the results obtained by the automatic method and the manual estimation (Figure 6a). It was also found that the agreement between results obtained by the two operators was also good (Figure 6b).

**Figure 7:**
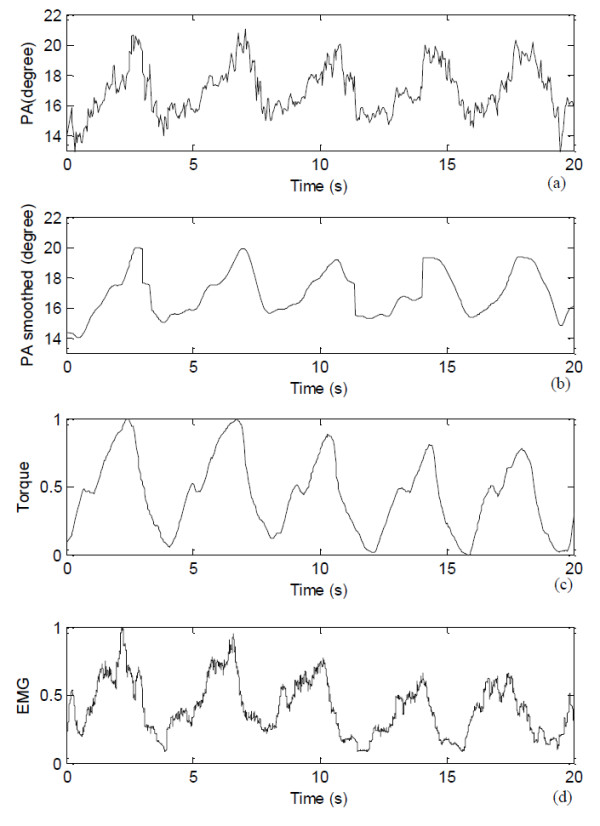
**Pennation angle along with the torque and EMG signals.** (**a**) The signal about the pennation angle (PA) estimated using the automatic method, (**b**) Smoothed signal of (**a**), (**c**) the torque signal recorded, and (**d**) the EMG RMS signal.

The pennation angle estimated using the automatic method was shown in Figure 7a together with its smoothed version (Figure 7b) which was processed using a smoothing algorithm proposed by Perona and Malik [22]. The corresponding torque signal and the root mean square (RMS, 256-points) values of EMG signals were shown in Figure 7c-d. The results showed that the signal about the pennation angle change well represent the cyclic contraction of the muscle, and this new signal can be used for functional assessment of muscles.

## Discussion

In this study, we separated the process for automatic estimation of the pennation angle into two steps, including the estimation of orientations of the deep aponeurosis and the muscle fascicles in a selected representative region. The muscle pennation angle can be obtained using the difference between these two orientations. A procedure was proposed for automatic measurement of pennation angle in a sequence of ultrasound images of muscles. Using the synthetic images with fascicle-like patterns with various noise levels, we demonstrated that the proposed fascicle orientation estimation method is robust.

In the estimation of the dominant orientation of the fascicles in the selected region of interested, we proposed to use the reliability of orientation field [20] to rule out contributions from regions where the texture orientation is not reliable or in other words, where the pattern appears more isotropic than oriented. The reliability coefficient ranges from 0 to 1, with 0 representing an isotropic region and 1 representing a strongly oriented pattern. When the reliability coefficient is smaller than a certain threshold, the calculated orientation is regarded as not reliable and should be neglected. In this study, we selected a threshold of 0.6, which was determined after many trials. Whether this value is applicable for ultrasound images from different muscles with different image qualities should be further investigated in the future.

It was noted that the original signal about the pennation angle changes detected using the proposed method was not as smooth as the torque signal, but was similar to the EMG RMS signal (Figure 7). This noisy feature was also observed in the pennation angle changes detected manually (Figure 5). The reason for such “noises” overlapped with the signals about the pennation angle changes was not clear, and future studies are required to better understand whether such “noises” are caused by intrinsic properties of muscle during contraction or by calculation errors. We have also demonstrated that the signal could be processed to become smoother. Using the original pennation angle changes, we compared the results obtained by the automatic method and the manual method, using the mean of the results obtained by the two operators. The results showed a good agreement (Figure 6) between the results by the proposed automatic method and the averaged manual estimation. Similar results have been reported previously [17,23]. The automatic method proposed in this paper may help solve problems of subjectivity and inconsistency caused by the conventional manual measurement, in addition to reduce the processing time.

## Conclusions

In summary, we have successfully developed a method for automatic measurement of muscle pennation angle in a series of ultrasound images of muscles. The preliminary results demonstrated the measurement was reliable. Further studies are required to test whether this new method is applicable for ultrasound images of different muscles under different contractions. Since the pennation angle can be easily obtained automatically using this new method, we think this important muscle parameter can be used more widely for the functional assessment of muscles.

## Competing interests

The authors declare that they have no competing interests.

## Authors’ contributions

YJZ: proposed the idea together with YPZ, JZL: analysed the data and composed the manuscript together with YJZ, GQZ: performed experiments together with YPZ and YJZ. All authors read and approved the final manuscript.
